# Aging as “Time-Related Dysfunction”: A Perspective

**DOI:** 10.3389/fmed.2020.00371

**Published:** 2020-07-27

**Authors:** Marios Kyriazis

**Affiliations:** National Gerontology Centre, Larnaca, Cyprus

**Keywords:** definitions of aging, clinical aging, hormesis, time-related dysfunction, health and function

## Introduction

Those of us who are involved in providing clinical services to older people, are in need of a clear definition of the term “aging” in order to provide better care. Biological or demographic definitions of aging do not, as a rule, take into account the every-day, patient-oriented aspect of aging, and are inadequate in reflecting the true nature of the patients' needs. When our aim is to work toward achieving better health in an older person, we need to take into account not only clinical, pharmacological or psychological issues, but we need to consider each patient as an individual, functioning in his/her own specific environment. In addition, all other aspects of health must be considered in a holistic manner, including sexuality, digestion, circulation, cognition, physical power, social matters etc.

Therefore, in my view, a step in the right direction, which may help health practitioners work toward improved health for an older person is to define aging as “Time-related Dysfunction.”

### Definitions of Aging

There have been many definitions of aging (1), each one depending on the specialty or interest of the researcher (Table 1).

**Table 1:** Some interpretations of aging.

1.	An inevitable progressive deterioration of physiological function with increasing age, demographically characterized by an age-dependent increase in mortality and decline of fecundity.	(2, 3)
2.	A decline or loss of adaptation with increasing age, caused by a time-progressive decline of Hamilton's forces of natural selection. Or, a persistent decline in the age-specific fitness components of an organism due to internal physiological degeneration.	(4)
3.	The biological process of growing older in a deleterious sense, what some authors call “senescence”.	(5–7)
4.	The intrinsic, inevitable, and irreversible age-related process of loss of viability and increase in vulnerability.	(8, 9)
5.	A failure of selection, due to either pleiotropic constraints or declining strength of selection after the onset of reproduction.	(10)
6.	Accumulation of damage over time.	(11)
7.	An accident of imperfect selection, where selection fails to purge deleterious, age-related mutations from an otherwise potentially immortal genotype.	(12, 13)

These are not incorrect definitions and there is no conflict between them, but they provide little help to the health practitioner in the clinic, or when dealing face-to-face with an older person. The plethora of biological, demographic or statistical definitions, and the lack of definitions which may help a health-care practitioner in practical terms is obvious, and necessitate another approach which is discussed here.

### A New Definition

A more relevant and useful method in this respect is to define aging as “Time-related dysfunction” (14). This definition implies that, with the passage of time and for a variety of causative factors, humans are subjected to damage which is not properly repaired. As a consequence, there is degeneration and loss of utility at all levels (molecular, cellular, tissue, organismic, and societal) with a resulting failure of the normal function of a human. In other words, it is a chronologically-depended erosion of our functions, which makes it increasingly difficult for us to manage and operate within a given, always-changing environment.

A consequence of this definition is the shift of emphasis from the “health/disease” aspect, to the “functional” one. To put it differently, the individual is seen in the context of a personalized environment, and what truly matters is the ability of that person to overcome tasks and challenges, irrespective of whether the person has a certain disease. The fact I attempt to convey here is that, in everyday life, it is the functional capabilities of the person that define the life of an older individual, capabilities that are matched to the specific person's internal and external milieu. It becomes irrelevant if a person has a diagnosed disease, a chronic issue, or a medical problem. What matters is whether this person is able to conduct everyday activities in a satisfactory and appropriate manner, matched to that person's aims and needs.

WHO defines Healthy Aging “as the process of developing and maintaining the functional ability that enables well-being in older age.” However, here I am not attempting to define Healthy Aging, but “Unhealthy Aging” in terms of degeneration which causes illnesses. Well-being may not always be relevant if the person remains creative and functional for long periods in their life.

### Clarifications

Here I am referring to dysfunction that it is caused by the passage of time (all the degeneration, processes, and biological mechanisms that fail due to age). I further explain it as “a chronologically-dependent erosion of our functions.” The main root cause of the dysfunction is the mere passage of time, which downregulates the repair mechanisms, increases the likelihood of disease, and thus causes increased probability of death. In children or teenagers there are no dysfunctions which are solely due to the passage of time. I am talking in broad terms, in the normal everyday sense of the words. If a condition is not related to the passage of time (for instance, a viral infection, injury, asthma), and if it does not affect function, then it should not generally be described as “aging.”

My intention is to describe aging in terms of functionality, because I am referring to real patients whose purpose in life is to function well within the limits of the human body. Aging is more than functionality, but from the aging patient's point of view, all that matters is function (for instance, the patient may have pain from arthritis but still be able to walk satisfactorily, i.e., function well-despite the pain, and despite the physical limitation).

## Discussion

This definition focuses specifically on real people in ordinary clinical situations. It places emphasis on function rather than on health, and thus bypasses complex biological, statistical, biodemographic or other aspects of aging, without claiming that these are wrong. It also bypasses notions of “looking young,” beauty or youthfulness. A person's life is defined by their normal operation within society, and we need to shift the emphasis from the current health-oriented, youth-oriented, or death rate-oriented aspects of aging, to a more pragmatic approach based on the value of each individual within society.

As mentioned above, the person could be considered “healthy” irrespective of the possible simultaneous presence of any disease, if that person is able to operate reasonably within his/her given environment. This environment may constantly, gradually or suddenly change, and the person should be able to continually adjust to these physical, psychological or social changes, in order to maintain adequate performance that matches those changes. If the person has the capability to modify their daily behavior in order to live reasonably within their sphere of abilities, without significant restrictions and without being overwhelmed, then this person is healthy and is aging well. Older people are a diverse group of different people who need personalized and individually-tailored approaches. With this definition of aging, it is not the degree of age-related degeneration that matters, but the ability of each person to respond and adapt to this degeneration. It is their individual ability to meet any challenges, and harmonize their life to match a constantly changing set of daily parameters (15).

Thus, healthy aging is the capacity to create positive environments and opportunities, in order to enable older individuals to meet their values and aspirations in a reasonable manner. The achievement of these values may not be at a 100% level, nevertheless even lower levels of achievement, say at an 80% achievement, may still ensure a reasonable standard of living, and the person can still be labeled as “healthy.”

There are several criteria and markers for assessing function in multiple domains (16). These should apply to each patient within their own situation, giving an individualized and not a general score. For example, these are some of the available tools:

* Barthel Index for Activities of Daily Living (17).
* The Functional Independence Measure (18).
* Six Minute Walk Test (19).
* Mini Mental Evaluation (20).
* Standardized Form-36 (21).
* Community Integration Questionnaire (22).

Thus, function may be measured and interventions to improve function can then be planned. Such interventions may include pharmacological, psychological or physical approaches, as well as addressing social issues, such as ageism, and improving targeted public services.

One way of working toward enhancing useful clinical function is to base our preventative or treatment methods on hormetic mechanisms. Hormesis is a well-studied phenomenon, where a low dose of a given stressor (or a challenge) may result in beneficial health effects by invoking a stress response, which up-regulates defense and repair pathways, whereas a higher dose of the same stressor may result in damage. It is a non-linear dose-response process (23). This may result in overall improvement of clinical function, and there is a myriad of specific methods that may invoke hormesis (24–27). Hormetic interventions broaden and expand the older person's limits of stress resistance, for example through expanding their Homeodynamic Space, the space where a person is able to adapt to challenges and survive (28).

A related and more specific approach is to encourage more cognitive activities instead of focusing mainly on physical ones (25, 29, 30). I have suggested (31) that increased cognitive positive (hormetic) stress acts through the neuronal stress response which in turn shifts repair resources from the germline to the neurons, thus improving age-related damage and ameliorates overall function. Thus, hormesis may enhance a person's ability to respond to any challenge originating from age-related degeneration.

I have highlighted that each person should be considered in relation to the environment she/he is in. If the needs or challenges originating from this environment are matched appropriately to the available functional abilities of the individual, then the person may be considered as “healthy” (32), and “not aged.” Aging therefore may be also seen as a mismatch between the challenges (biological, medical, social, psychological, technological, cultural) facing a person, and the available resources to overcome those challenges, in order for the individual to continue operating well. It is also worth highlighting that the capacity to overcome challenges is specific to each individual, at any given time, at any given environment, and it is not the same for every age group or for every person.

## Conclusions

I am arguing that health in later life is not only dependent on well-being but on function. It may not even depend on other commonly used notions such as vitality, vigor, physical or mental strength etc., as long as the person operates well within the sphere of their needs. My suggestion is that health practitioners should promote strategies which ensure high levels of functioning across several domains, including physiological, psychological, emotional, technological and societal. This fosters improved resilience and facilitates adaptation to the many challenges facing older people today (33–36).

In this viewpoint, the consideration of aging not as a matter of health, but a matter of function eliminates the unnecessary need of treating each medical condition at all costs (with all the associated risks of adverse effects, iatrogenic diseases etc.), but facilitates a smoother two-way process between the patient and his/her ever-changing, specific and personal needs. Of course, treating and attempting to cure each disease does indeed help in the overall operating of the patient, but it is not necessarily essential. For instance, a person who has a certain weakness due to stroke, may well be able to continue their life as normal, as long as they adjust their aims and aspirations in order to match that weakness. Existing definitions of the term “aging” therefore become not relevant, although not incorrect. In my view, aging as a “Time-related dysfunction” is a more appropriate term. The aim should not necessarily be to cure someone from their illness, but to find ways to improve their function so that they can contribute successfully to society.

## Author Contributions

The author confirms being the sole contributor of this work and has approved it for publication.

## Conflict of Interest

The author declares that the research was conducted in the absence of any commercial or financial relationships that could be construed as a potential conflict of interest.
